# The Metropolitan Asylums Board of London and Its Work

**Published:** 1900-10-13

**Authors:** 


### THE METROPOLITAN ASYLUMS BOARD OF LONDON AND ITS WORK.

### II.—STATE-SUPPORTED CHILDREN.

In a recent article on the work of the Metropolitan Asylums Board of London, brief reference was made to that department which concerns itself with "the education and training of certain classes of children who become chargeable to the State." It may be interesting to review rather more in detail the very important work which is being now done in this direction through the instrumentality of the Board.

To go back to past history, before the year 1834, those children who came under the jurisdiction of the Poor Law were not separately provided for, but were housed in the workhouses with the adult paupers, an arrangement as pernicious in its effect on these young people as can well be imagined. The first step towards improvement came with the establishment of voluntary institutions, where the children were boarded out by the Guardians of their respective parishes, an arrangement undertaken, a little later, entirely by the Poor Law authorities themselves. To minimise expense various metropolitan unions were combined into districts, so that one school has served two or more parishes, large buildings, commonly known as "barrack schools," which speedily proved more or less unmanageable in several respects. To quote the report: "Classification is never easy except in a colony composed of numerous small buildings, and in a school composed of a few very large buildings, as most of these were (and as, indeed, all but two now are), classification became very difficult indeed. The most noticeable result was that when an infectious or contagious disease was introduced into the school it spread rapidly. The difficulties attending outbreaks of an infectious disease, such as scarlet fever, have been largely met by the provision of isolation hospitals in the school grounds, but so far as contagious diseases are concerned (ophthalmia, for instance), not much has or can very well be done. Consequently outbreaks of ophthalmia, ringworm, and other eye and skin diseases were not infrequent." The prevalence of such outbreaks formed the subject of many reports and investigations, noticeable amongst these being the report issued by Mr. Nettleship, at the request of the Local Government Board, in 1874. In 1896 the many defects proved by experience to attach to the barrack system of schools formed the subject of an investigation by a committee appointed to inquire into the management of Poor Law schools. The report of this committee recommended that such schools should be composed of small rather than large buildings, with the object of imitating in small houses, as far as possible, the ordinary home life of ordinary children. It was presently proposed to create a new body to deal with exceptional children, but ultimately this work was handed over to the Asylums Board, the classes of children under their care being thus specified in an order from the Local Government Board, dated April, 1897:—

(*a*) children suffering from ophthalmia or other contagious disease of the eye;

(*b*) children suffering from contagious disease of the skin or scalp;

(*c*) children requiring either special treatment during convalescence or the benefit of seaside air;

(*d*) children who by reason of defect of intellect or physical infirmity cannot properly be trained in association with children in ordinary schools; and

(*e*) children who are ordered by two justices or a magistrate to be taken under the Industrial Schools Act, 1866, to a workhouse or an asylum of the district.

Thus it is seen that under this new arrangement "exceptional children" are removed from the ordinary Poor Law schools, to the obvious benefit of themselves and of the normal children left behind, and are accorded that special medical care and treatment which they require, and the education adapted to their individual peculiarities

It is estimated that of the 18,000 children under the London Poor Law some 2,000 will be under the care of the Managers of the Board, totalling up as follows:—Ophthalmia, 800; ringworm, 400; convalescents, 360; defective children, 360; remand children, 150.

Now, let us see what steps the Board has taken to meet the responsibilities thus placed upon them. The first efforts of the Managers were directed towards the provision of proper accommodation for children suffering from ophthalmia (the largest class with which they had to deal), and two sites have been purchased, one in Kent and the other in Essex, whereon schools are being erected on the following plan. There will be thirty cottages, each cottage holding ten children, under the charge of a "house-mother," the cottages built in pairs, and every three pairs supplemented by a fourth cottage, for cooking purposes, and where the charge-nurse will attend to the children's eyes, and have her apartments. Ample accommodation in the way of laundries, isolation cottage, &c., is arranged for.

With regard to children suffering from ringworm, the Managers have arranged to purchase a school at Sutton from the dissolving South Metropolitan School District Board, which will be set apart for their special use.

For convalescents, two seaside homes, already established by Guardians, were acquired by the Board, and two others have been taken over at Herne Bay and at Margate, while at the latter place two more houses will be built, and three others on the South Coast, at Rustington, for cases needing milder air. The attention of the Board has also been directed to the question of dietary and a more elastic system established, providing for greater variation in detail than obtains in ordinary Poor Law establishments. It has been wisely decreed, too, that the children will not be marked out by special uniform, but clothed as the various matrons may decide at their discretion.

Then we come to "defective children"—*i.e.*, cases of feeble mind, and of blindness, deafness, partial paralysis, &c. To meet the requirements of such children the Managers decided to take small houses in convenient districts of London, near the special schools provided for such cases by the London School Board. Two houses have been purchased and negotiations are in progress for others.

Finally, the Board has had to arrange for "remand children," those who, having been charged before a magistrate for offences which render them liable to be sent to an industrial school, have been remanded. These poor little sinners are in this way rescued from pauper contamination in the workhouses, to which they have hitherto been consigned pending inquiries. Three houses have been secured and will soon be open for the reception of these children, who will there be suitably dealt with and protected from the poisoning knowledge of prison or pauper life.

The Metropolitan Asylums Board has, it will be seen, embarked upon a heavy and responsible task; the success of the various schemes evolved to meet the growing needs of the vast population of London will be looked for with deep and general interest.

